# CAUSES OF FRAILTY: GENETIC ANALYSIS IN 164,000 UK BIOBANK PARTICIPANTS

**DOI:** 10.1093/geroni/igz038.812

**Published:** 2019-11-08

**Authors:** Janice Atkins, Luke C Pilling, Dylan Williams, Juulia Jylhävä, David Melzer

**Affiliations:** 1 University of Exeter, Exeter, Devon, United Kingdom; 2 University of Exeter, Exeter, England, United Kingdom; 3 Karolinska Institutet, Stockholm, Stockholms Lan, Sweden

## Abstract

Frailty is an important and common geriatric syndrome, yet the mechanisms are multifactorial and not well understood. The Frailty Index (FI) is based on the Rockwood Index for accumulation of deficits, and we aimed to use genetics to gain mechanistic insights for interventions to prevent and delay frailty in older people. We performed a genomewide association study in 60 to 70 year old UK Biobank participants of European descent (n=164,610). We identified 26 independent genetic signals at 24 loci associated with the FI. Several of these signals have previously been associated with traits such as cardiovascular disease, intelligence, and educational attainment, but 6 of the signals appear to be novel. We will also present the results of ongoing work both to identify causal risk factors for FI using Mendelian randomization methods, and replication and functional follow-up in the TwinGene cohort, at the meeting.

